# The Use of Artificial Intelligence in Palliative Care Communication: A Narrative Review

**DOI:** 10.7759/cureus.80524

**Published:** 2025-03-13

**Authors:** Andrea Pinto, Catarina Santos, Rita Aguiar, Sara Oliveira, Daniela Cunha

**Affiliations:** 1 Nursing, Hospital Dr. Nélio Mendonça, Funchal, PRT; 2 Medical-Surgical Nursing, Escola Superior de Saúde Norte da Cruz Vermelha Portuguesa, Oliveira de Azeméis, PRT; 3 Nursing, Instituto Português Oncologia do Porto Francisco Gentil, Porto, PRT; 4 Nursing, Centro Hospitalar de Entre Douro e Vouga, Santa Maria da Feira, PRT; 5 Nursing, Unidade Local de Saúde de Coimbra, Coimbra, PRT

**Keywords:** artificial intelligence, communication, decision making, forecasting, palliative care

## Abstract

Technological advances, such as "machine learning" and "natural language processing," have enabled systems and machines to perform complex tasks that previously required human intervention. Artificial intelligence (AI) has emerged as one of the most significant advancements in the healthcare sector, playing a key role in the evolution of palliative care (PC). Our main objective was to explore how AI can improve the quality of communication in decision-making in PC.

A narrative review was conducted to obtain an interpretative synthesis and a comprehensive perspective on the subject under analysis. The research was carried out using the terms "Palliative Care," "Communication," "Artificial Intelligence," "Forecasting" and "Decision Making."

Nine articles were included in the study, and after data analysis under Jean Watson's Theory of Transpersonal Caring, four categories were defined that respond to the proposed objective: person-centred care and authentic relationships, decision support based on individualised knowledge, facilitation of transparent communication and advanced care planning, promotion of a healing environment and emotional well-being and education of health professionals and critical reflection. As a result, we identified the need for a multifaceted approach, involving the continuous validation of models, proper training of healthcare professionals and engagement of individuals in decision-making processes. This ensures that decisions are grounded in robust evidence and ethical principles, making sure that AI acts as a true ally rather than a source of additional risks.

In conclusion, AI can effectively be a valuable support tool in decision-making, but it is crucial that professionals remain aware of its limitations and can apply critical judgment in each situation.

## Introduction and background

Palliative care (PC) promotes a therapeutic approach that aims to provide comprehensive support to people with life-limiting illnesses, focussing on symptom relief, improving quality of life and meeting the emotional, psychological and spiritual needs of people and their families [1].

PC is part of a multidisciplinary approach aimed at minimising suffering and offering comfort to the patient and family. Early access to PC promotes emotional well-being, better coping strategies, reduced health costs and contributes to quality of life [1]. 

Artificial intelligence (AI) is one of the most significant advances of the digital transformation in the health sector, playing a key role in PC. AI is defined as the ability of a machine to perform tasks that normally require human intelligence, including reasoning, learning, perception and decision-making [2]. Artificial systems such as machine learning (ML) and natural language processing have enabled the creation of conversational agents designed to support activities in healthcare, including treatment, health monitoring, triage and screening [3,4]. 

In the context of PC, AI can improve communication between healthcare professionals, individuals and families, provide information in real time and enable the personalisation of approaches according to the needs of each individual [5]. Natural language processing and ML have demonstrated their ability to facilitate the understanding and transmission of complex clinical information [4].

AI can help identify communication patterns that promote empathy and emotional support, especially during the sharing of difficult information, such as end-of-life information [6]. These systems are able to recognise emotional signals and adapt the tone of the conversation according to the person's emotional state, helping professionals to maintain an empathetic and humanised approach, even in critical moments.

However, the use of AI in PC faces important challenges. The humanisation of care is a fundamental principle of PC, and the ability to maintain authentic and empathetic communication remains an area where artificial systems cannot replicate the complexity of human interaction [7].

Conversational agents can offer personalised information, answer questions and provide emotional support, as well as helping to monitor and manage symptoms [4]. However, these systems are complementary tools and do not replace direct interaction between the healthcare professional and the person, which should always be maintained and strengthened [2]. 

AI can provide clear information, facilitating decision-making processes, but it must not interfere with the autonomy of individuals or their right to informed consent. It must respect ethical and bioethical boundaries, ensuring that decisions about care follow the person's wishes and values [5,6].

Predictive AI models have been studied to identify people who benefit from PC, reducing response time and increasing the likelihood of early appropriate interventions, improving quality of life. These models analyse large volumes of clinical data, identifying patterns that predict disease progression and the need for specific care [1]. 

The regular implementation of AI is crucial to guarantee privacy, information and informed consent. AI can improve the quality of life of people undergoing PC, optimise decision-making processes and provide a more personalised and efficient approach. However, this implementation must be accompanied by critical reflection on the ethical and bioethical challenges, ensuring that care remains person-centred and does not compromise the therapeutic relationship that is essential to the practice of care [6]. 

Jean Watson's Transpersonal Care Theory is an appropriate choice for studying AI in PC. Watson emphasises that nursing care involves spiritual, cultural and existential dimensions, which are essential for humanised, person-centred care [8,9]. AI should be a complementary tool that can improve clinical accuracy and decision-making, but which does not replace human contact or the genuine interaction that is fundamental in PC. In light of Watson's theory, this technology must respect and value people's individuality and values, ensuring that care follows principles of dignity and compassion [10]. Watson also recognises the importance of emotional and spiritual support in situations of great vulnerability, such as critical moments in PC [11]. 

Using AI in PC communication can transform the care provided by personalising communication, improving emotional support and speeding up access to relevant information. This study explores the use of AI in PC communication to improve the quality of care provided by personalising communication, improving emotional support and speeding up access to relevant information, promoting better interactions between the person/family and healthcare professionals.

The results suggest that AI can facilitate more informed and personalised clinical decisions, using technological tools to predict clinical failures and adjust care to individual needs [12,13]. In addition, the results of this research can contribute to improving communication during critical moments, such as discussions about prognosis, therapeutic options, and end-of-life care preferences.

AI can be a supportive tool, making conversations clearer, grounded in scientific evidence and aligned with individuals' values, easing the emotional burden and reinforcing trust in decision-making processes [14].

## Review

Methodology

A narrative review was carried out in order to obtain a comprehensive perspective and provide an interpretative synthesis on the subject under analysis. This methodology allows for the expeditious integration of different sources but does not allow for an exhaustive mapping of the literature, and the selection of articles is conditioned by the authors' assessment of their relevance to answering the research question: “How can the use of AI improve the quality of communication between health professionals, people and their families in PC decision-making?”

A narrative literature review was carried out in the CINAHL Complete, MEDLINE Complete, Nursing & Allied Health: Comprehensive Edition, Cochrane Controlled Trials Register, Cochrane Database of Systematic and Medic Latina databases, using the terms “Palliative Care,” “Communication,” “Artificial Intelligence,” “Forecasting” and “Decision Making,” in Portuguese, English and Spanish.

Using Boolean operators, the following search equation was formulated: “(palliative care AND artificial intelligence) AND (nursing care OR communication OR decision making OR forecasting)” in the three languages chosen. 

The studies were selected based on the following inclusion criteria: (1) studies investigating the use of AI in PC; (2) studies related to safety in the use of AI; (3) studies addressing the use of AI in healthcare decision-making.

The exclusion criteria were: (1) studies that did not present specific results on AI; (2) studies involving people under the age of 18 or the paediatric population; (3) studies that were not available in full-text format.

The selected articles were subjected to a full-text review. For each study included, the following data were extracted: authors, year of publication, country of conduct, type of study, objectives, population analysed and main conclusions.

Results 

To answer the study question, nine articles were included; the limited research on this subject focussed on the emergence of AI in the context of PC. The majority took place in the United States of America (USA), totalling 55.6%. The studies analysed have significant differences in the representativeness of the groups involved in PC. The most represented were patients with neoplasms (22.2% of the total). The results are summarised in Table 1.

**Table 1:** Summary of results from the included studies AI: Artificial Intelligence; ML: Machine Learning; NA: Not Available; PC: Palliative Care

S. No.	Authors	Year	Title	Type of study	Objective(s)	Population	Sample size	Main conclusions
1	Cagliero et al. [15]	2023	A framework to identify ethical concerns with ML-guided care workflows: a case study of mortality prediction to guide advance care planning	Case study	Identifying ethical concerns with ML applications to healthcare	Stakeholders interviewed	70	ML Integration: Disagreement over whether to introduce ML results into the clinical process at critical moments. Distribution of Predictions: Disagreement over who should receive predictions (clinicians, patients and family members). Benefits and Risks: Discussion over the balance between benefits and potential harms of predictions in sensitive care. Fiduciary Responsibility: Discussion over the AI ​​team’s responsibility to patients and healthcare professionals. Research Protection: Need to protect initial research from external influences until trials are completed. Machine Learning Team Priorities: Develop alternative strategies for implementing ML; Clarify that predictions are intended for advance care planning; Protect research from external pressures during trials; Identify and address relevant ethical tensions in implementing AI in PC.
2	Matt et al. [16]	2023	An acoustical and lexical ML pipeline to identify connectional silences	Cohort study	A pipeline of ML algorithms to automatically identify and subclassify Connectional Silences in natural clinical settings	Hospitalised persons with advanced cancer, their families (if present) and palliative care specialists	285	Connectional Silence in PC: Associated with positive outcomes such as improved quality of life and treatment decisions aligned with the patient’s preferences. Data Collection Context: Each conversation was recorded with a portable omnidirectional device placed unobtrusively in the patient’s room; Identifying information was removed from the audio files. ML Pipeline: Performed well in identifying moments of Connectional Silence, with high specificity in categorising the identified pauses. An ML pipeline that integrates different acoustic and lexical representations can automatically identify moments of Connectional Silence in natural clinical conversations about serious illness. Future Strategies: Obtain equitable, meaningful, and scalable conversational analytics for large samples in research and quality improvement.
3	Srivastava et al. [17]	2023	Can artificial intelligence aid communication? Considering the possibilities of GPT-3 in palliative care	Case study	Understand the possibilities of AI-aided communication in palliative care	Psychologists with experience in palliative care	6	Conversations in Palliative Care: Conversations about palliative care become crucial after a terminal diagnosis. However, healthcare professionals often feel inadequate when addressing topics related to death and the dying process. AI Technologies in Mental Health: Several chatbots and AI technologies have been developed to provide mental health support and advice. Simulation of Human Conversations: AI uses natural language processing and machine learning algorithms to simulate human-like conversations, offering emotional support. Limitations of AI in Palliative Care: The use of AI in palliative care is limited. Although it may appear courageous, it is often naive, hasty or even vain when addressing sensitive topics. Difficulties in Therapeutic Conversations: Conversations with AI, such as GPT-3, for therapeutic and decision-making purposes in palliative care present difficulties, such as: Artificial Empathy - Limitation in generating genuine empathy; Emotional Impact - Lack of sensitivity to the emotional impact on people; Cultural Differences - Insensitivity to cultural variations; Lack of Human Judgement - Inability to exercise judgement and intuition like a human being. Role of Advanced AI: While technologies like GPT cannot replace humans in communication and guidance in PC, they have the potential to act as conversational experts.
4	Schenker et al. [18]	2024	Conversational agents in palliative care: potential benefits, risks and next steps	Review article	Explore conversational tools for improving patient and family outcomes in serious illness	Agents in healthcare	NA	PC and AI: Many consider PC, which focuses on human connection, to be incompatible with AI-based conversational agents. Advances in AI: Machine learning and natural language processing have increased the use of conversational agents in healthcare. Role of Conversational Agents: Can address emotional and psychological needs, monitor physical symptoms, provide non-pharmacological interventions and serve as spiritual counsellors or virtual companions. Care Coordination: Can facilitate coordination and referral to specialised care, but do not replace clinicians. Potential Risks: Transparency, data security and privacy, harm, bias and inequality in access. Risk Mitigation: Requires multidisciplinary teamwork involving experts in healthcare, technology, data science and communication.
5	Petersson et al. [19]	2023	Ethical considerations in implementing AI for mortality prediction in the emergency department: linking theory and practice	Qualitative study	Explore healthcare professionals' perspectives on ethical issues in AI implementation for predicting patient mortality in emergency departments. Develop a model based on ethical theory to guide ethical considerations in AI implementation in healthcare.	Healthcare workers in the emergency departments	18	Challenges in PC: Shortage of workers, increasing demand, high costs, scarcity of resources and inequality in care provision; Improvement facilitated by digitalisation with AI. Human Factors: The introduction of AI in healthcare raises ethical issues, systemic challenges and implications for professional routines and responsibilities. Ethical Framework: Six ethical principles (autonomy, beneficence, non-maleficence, justice, explainability and governance) should be considered according to ethical theories in the implementation of AI. More research is needed to guide the ethical issues of applying AI in healthcare.
6	Strechen et al. [20]	2024	Harnessing health information technology to promote equitable care for patients with limited English proficiency and complex care needs	Randomised trial	Assess the effectiveness of comprehensive intervention, integrating artificial intelligence with a human operator into the language services process to provide in-person interpreters to patients with complex care needs	Individuals who have a non-English language preference	35	Impact of AI in PC: Study on the incorporation of machine learning algorithms to improve the provision of interpreter services to inpatients with complex needs. Monitoring and Feasibility: Continuous monitoring of the effectiveness and feasibility of integrating AI into the clinical workflow. Study Context: Conducted in multiple hospital settings, including ICUs and emergency departments. Advantages: Reduced cross-contamination among clinicians and gradual introduction of interventions, reflecting real practices and ensuring feasibility. Integration Gaps: Significant challenges remain in integrating and evaluating algorithms in real clinical settings.
7	Hiratsuka et al. [21]	2023	Prediction of survival in patients with advanced cancer: a narrative review and future research priorities	Review article	Summarise the current situation of prognostication for patients with an expected survival of weeks or months, and clarify future research priorities	Palliative care clinicians	NA	Importance of Prognostic Information in End-of-Life Care: Prognostic information is essential for individuals, their families and healthcare professionals to make decisions about end-of-life. Life expectancy directly affects care, influencing decisions such as systemic anticancer treatment and end-of-life. Lack of Consensus: There is no consensus on the most appropriate methods to compare tools with different survival prediction formats. Future Research Priorities: Importance of communicating prognosis in end-of-life discussions and advanced care planning; Impact of understanding the disease trajectory on individuals: facilitates discussions about goals of care, important decisions and end-of-life preparation; promising role of AI in predicting unexpected death, not covered by traditional prognostic models, through the creation of complex models with large demographic, laboratory, imaging and genetic databases. AI in Professional Education: Using prognostic calculators to recognise personal biases of healthcare professionals. Further studies are needed to assess whether education improves confidence and accuracy in prognostication in PC. Prognosis implies uncertainty, and a standardised training program is needed to improve practices.
8	Reddy et al. [22]	2023	Recent advances in artificial intelligence applications for supportive and palliative care in cancer patients	Literature review	Provide an overview of the recent studies applying AI to support palliative care patients	Cancer patients	29	AI in PC: AI uses computing to process large volumes of data and has the potential to improve PC, although it is still in its early stages. Mortality Prediction Models: Reviews show that AI is effective in predicting short-term mortality, especially through “machine learning” and “deep learning.” These models help identify people at high risk, improving the discussion of goals of care and facilitating interventions in advanced care plans. Concerns About AI: There are concerns about bias, lack of reproducibility and negative perceptions that can generate anxiety and stress in people. Prediction of Other Clinical Events: AI can predict other events, such as lung disease and hospitalisations, allowing for more intensive monitoring and reducing emergency room visits. Support Needs and PC: AI can predict the need for PC, quickly identifying people who would benefit from it. Text Triage Models: AI can identify people’s symptoms and conditions, highlighting those with urgent needs or without an advanced care plan, and analyse support group conversations, monitoring emotional well-being for more empathetic care.
9	Burry et al. [23]	2024	‘‘You are not alone”: the allure and limitations of artificial intelligence in serious illness communication	Review article	Exploring the allure of employing AI-powered chatbots to assist nonspecialist clinicians with serious illness communication and highlights the ethical and practical draw backs.	Nonspecialist clinicians	NA	Difficult Conversations in PC: Healthcare professionals often face difficult conversations when caring for people with serious illnesses, including delivering bad news and setting care goals. Necessary Training for Non-palliative Specialists: Generalists and specialists in non-palliative care, who facilitate most conversations about serious illnesses, need adequate training. AI as a Solution for Inexperienced Professionals: AI can be a useful tool for professionals with less training, assisting in communication. Limitations of AI in Communication: AI uses words in a formless manner, does not convey non-verbal communication or silence and does not understand the emotional interaction demonstrated by people and caregivers. Uncertain Future of AI in PC: Although the role of AI in the future is uncertain, it currently does not replace communication skills in palliative care. Potential to Increase Confidence: AI can be useful in boosting the confidence of inexperienced professionals in difficult conversations by suggesting phrases and creating a logical structure to guide communication.

Jean Watson's Transpersonal Care Theory categorises the data according to its central elements and emphasises holistic care aimed at the integrality of the person. The first category is Centred Care and the Authentic Relationship, which establishes a natural connection between health professionals and the person, considering emotional, spiritual and cultural needs. Information analysis can help communicate and organise information, allowing healthcare professionals to devote more time to direct interaction and therapeutic presence. This can reduce bureaucratic time and provide more opportunities for emotional support [10].

The second category is Decision Support based on Individualised Knowledge, which tailors care to each person's needs and preferences. AI can compile and analyse large volumes of data, offering personalised predictions that support clinical decisions and better care planning and facilitate informed, transparent and assertive communication, respecting Watson's principles of beneficence and autonomy [11]. 

According to Jean Watson, clear communication and explanation are essential for effective treatment. AI can simplify complex medical terms and provide materials for diagnosis and treatment options, supporting early care planning. It can also help structure difficult conversations, suggesting appropriate phrasing and guiding healthcare professionals in PC [24]. 

The fourth category is Promoting the Care Environment and Emotional Wellbeing, which emphasises the importance of the environment for emotional and spiritual growth. AI can monitor emotional symptoms and anxiety symptoms and analyse conversations and emotional well-being data, making it possible to create a more empathetic and personalised therapeutic environment in line with the promotion of emotional well-being advocated by Watson's theory [25].

Finally, Health Professional Education and Critical Reflection is a fundamental aspect of Watson's theory, advocating continuous professional development, including critical thinking and self-knowledge. AI can support the training of healthcare professionals, helping them to recognise patterns in their behaviour and providing feedback based on interactions. In this way, it contributes to the development of critical thinking and the improvement of clinical practice, in line with Watson's theory [11]. 

Therefore, the results were grouped into five categories for better understanding. The first category, Person-Centred Care and Authentic Relationships, highlighted issues related to the integration of AI into the clinical process. There were disagreements about introducing ML results at critical moments of care, as well as concerns about the distribution of forecasts - specifically, who should receive them: doctors, patients or family members. Additionally, the concept of “connectional silence” in PC was discussed, emphasising the importance of improving quality of life and ensuring that decisions align with the individual’s preferences.

In the second category, Decision Support Based on Individualized Knowledge, the discussion emphasized balancing the benefits and potential risks of predictions in sensitive care settings. The fiduciary responsibility of the AI team towards patients and healthcare professionals was also debated. Furthermore, AI's role in simulating human conversations was explored, with the goal of offering emotional support and improving decision-making in alignment with personal preferences. 

In the Facilitating Transparent Communication and Advance Care Planning category, we identified that the priorities of the ML team should include developing alternative strategies focussed on advance care planning while protecting research from external influences. The results highlighted the importance of an efficient ML pipeline, particularly in identifying moments of connectional silence and fostering an empathetic environment. In this context, the importance of integrating AI into care planning to enhance service delivery was also emphasised.

The results also address the Promotion of a Healing Environment and Emotional Well-Being. In this category, the discussion focused on future strategies to ensure equity in conversational analyses to support emotional well-being. However, the limitations of AI in this domain were acknowledged, particularly its inability to convey genuine empathy and capture non-verbal nuances essential for care. While advanced technologies such as GPT can assist in conversations, they do not replace the necessity of an authentic human presence.

Finally, in the Health Professional Education and Critical Reflection category, the results suggest that AI can play a role in training and supporting less experienced professionals by providing communication suggestions. However, ethical concerns were raised, including transparency, security and data privacy. The implementation of AI should adhere to an ethical model based on principles such as autonomy, beneficence, non-maleficence, justice, explainability and governance. Additionally, it was recognised that AI still struggles to generate empathy and understand the emotional impact of therapeutic conversations, reinforcing the need for human involvement in these interactions.

Discussion of results 

Analysing the studies reveals a significant disparity in the representation of the various groups involved in PC. While patients and their families are often included, other groups such as psychologists, emergency professionals and non-specialised clinicians are less represented. This imbalance in the groups studied impoverishes the holistic and multidisciplinary vision that is required in the PC approach. 

AI can help balance the benefits and risks of PC interventions. An example of this is predictive modelling, which helps to make early referrals to PC. However, its results depend on the reliability of the data input and require continuous supervision. This participatory approach ensures that care is aligned with each person's values and preferences, reinforcing the quality of the decisions made [6,26]. 

The promotion of an emotional environment and emotional well-being is another area that can benefit from the use of AI; although it cannot replicate empathy, it relieves workload and improves operational efficiency. As such, it is a complementary tool, not a substitute for human interaction. Clinical decisions should always be based on robust evidence and supported by a clear ethical understanding of the person's wishes and needs, ensuring that AI is a true ally and not a source of additional risk [2]. 

The main limitations found in this narrative review were the lack of rigour and systematisation of the studies found, namely in the selection of the study population and the evaluation of the studies. The absence of a strict protocol can lead to bias in the selection of articles, influenced by subjective judgements, resulting in confirmation bias. The lack of strict criteria for assessing the quality of the studies included compromised the robustness of the conclusions. In the specific case of our narrative review, the unequal representation of the different stakeholders stands out, with an under-representation of populations reducing the comprehensiveness and equity of the findings.

This narrative highlights the need for more rigorous and systematic methodologies in future research and future suggestions and guidelines for researchers and professionals have been defined, which will contribute to a more grounded and inclusive practice.

## Conclusions

AI use in PC has strengths and limitations, requiring a balance between technology and person-centred care while upholding ethical principles like beneficence, autonomy and privacy.

The appropriate use of AI depends on proper training and the integration of various stakeholders in care decision-making. The use of predictive models must undergo rigorous analysis to ensure that AI-based decisions remain flexible, contextualised and compatible with ethical principles.

## References

[1] Ferreira MP, Abejas AG (2023). Patient referral to palliative care using artificial intelligence prediction models. Lusíadas Sci J.

[2] Aydogdu A (2022). Artificial intelligence and nursing: reflection on the use of technologies in the caregiving process (Article in Portuguese). Revista de Enfermagem UFJF.

[3] Stamer T, Steinhäuser J, Flägel K (2023). Artificial intelligence supporting the training of communication skills in the education of health care professions: scoping review. J Med Internet Res.

[4] Milne-Ives M, de Cock C, Lim E (2020). The effectiveness of artificial intelligence conversational agents in health care: systematic review. J Med Internet Res.

[5] Dias P (2023). Use of artificial intelligence in diagnosing and determining eligibility for palliative care: ethical and legal limits (Article in Portuguese). Revista de Saúde Digital.

[6] Nunes HC, Guimarães RMC, Dadalto L (2022). Bioethical challenges of using artificial intelligence in hospitals (Article in Portuguese). Revista Bioética.

[7] Schenker Y, Arnold RM, Matt L (2023). Challenges of using artificial intelligence in palliative care: empathy and communication in end-of-life scenarios. J Palliat Med.

[8] Watson J (2002). Nursing: Human Science and Caregiving - A Nursing Theory (Book in Portuguese). Enfermagem: Ciência humana e cuidar - uma teoria de enfermagem.

[9] Watson J (2008). Nursing: The Philosophy and Science of Caring. University Press of Colorado, Boulder.

[10] Evangelista CB, Lopes ME, Nobrega MM (2020). Analysis of Jean Watson's theory according to the Chinn and Kramer model (Article in Portuguese). Revista de Enfermagem Referência.

[11] Riegel F, Crossetti MD, Siqueira DS (2018). Contributions of Jean Watson's theory to holistic critical thinking of nurses. Rev Bras Enferm.

[12] Ahmad O, Stanley S, Mason S (2021). Artificial intelligence in palliative care: a systematic review to identify its scope of use. BMJ Support Palliat Care.

[13] Utria-Munive J (2024). Incorporating artificial intelligence in palliative care: opportunities and challenges. Hospice Palliat Med Int J.

[14] Vu E, Steinmann N, Schröder C (2023). Applications of machine learning in palliative care: a systematic review. Cancers (Basel).

[15] Cagliero D, Deuitch N, Shah N, Feudtner C, Char D (2023). A framework to identify ethical concerns with ML-guided care workflows: a case study of mortality prediction to guide advance care planning. J Am Med Inform Assoc.

[16] Matt JE, Rizzo DM, Javed A (2023). An acoustical and lexical machine-learning pipeline to identify connectional silences. J Palliat Med.

[17] Srivastava R, Srivastava S (2023). Can artificial intelligence aid communication? Considering the possibilities of GPT-3 in palliative care. Indian J Palliat Care.

[18] Schenker Y, Abdullah S, Arnold R, Schmitz KH (2024). Conversational agents in palliative care: potential benefits, risks, and next steps. J Palliat Med.

[19] Petersson L, Vincent K, Svedberg P, Nygren JM, Larsson I (2023). Ethical considerations in implementing AI for mortality prediction in the emergency department: linking theory and practice. Digit Health.

[20] Strechen I, Wilson P, Eltalhi T (2024). Harnessing health information technology to promote equitable care for patients with limited English proficiency and complex care needs. Trials.

[21] Hiratsuka Y, Hamano J, Mori M, Maeda I, Morita T, Suh SY (2023). Prediction of survival in patients with advanced cancer: a narrative review and future research priorities. J Hosp Palliat Care.

[22] Reddy V, Nafees A, Raman S (2023). Recent advances in artificial intelligence applications for supportive and palliative care in cancer patients. Curr Opin Support Palliat Care.

[23] Burry N, Nakagawa S, Blinderman CD (2024). “You are not alone”: the allure and limitations of artificial intelligence in serious illness communication. J Palliat Med.

[24] Favero L, Meier M, Lacerda M (2009). Application of Jean Watson’s transpersonal caring theory: a decade of Brazilian production (Article in Portuguese). Acta Paul Enferm.

[25] Silva C, Valente G, Bitencourt G (2010). The transpersonal caring theory in nursing: analysis according to Meleis (Article in Portuguese). Cogitare Enferm.

[26] Rechmann IL (2024). Reflections on the use of artificial intelligence in the care of oncology patients (Article in Portuguese). Direito UNIFACS.

